# A multiple biomarker assay for quality assessment of botanical drugs using a versatile microfluidic chip

**DOI:** 10.1038/s41598-017-12453-w

**Published:** 2017-09-25

**Authors:** Zhen-Hao Li, Ni Ai, Lawrence X. Yu, Zhong-Zhi Qian, Yi-Yu Cheng

**Affiliations:** 1 Pharmaceutical Informatics Institute, College of Pharmaceutical Sciences, Zhejiang University, Hangzhou, China; 2 Center for Drug Evaluation and Research, Food and Drug Administration, Silver Spring, USA; 3 Tianjin State Key Laboratory of Modern Chinese Medicine, Tianjin University of Traditional Chinese Medicine, Tianjin, China

## Abstract

Quality control is critical for ensuring the safety and effectiveness of drugs. Current quality control method for botanical drugs is mainly based on chemical testing. However, chemical testing alone may not be sufficient as it may not capture all constituents of botanical drugs. Therefore, it is necessary to establish a bioassay correlating with the drug’s known mechanism of action to ensure its potency and activity. Herein we developed a multiple biomarker assay to assess the quality of botanicals using microfluidics, where enzyme inhibition was employed to indicate the drug’s activity and thereby evaluate biological consistency. This approach was exemplified on QiShenYiQi Pills using thrombin and angiotensin converting enzyme as “quality biomarkers”. Our results demonstrated that there existed variations in potency across different batches of the intermediates and preparations. Compared with chromatographic fingerprinting, the bioassay provided better discrimination ability for some abnormal samples. Moreover, the chip could function as “affinity chromatography” to identify bioactive phytochemicals bound to the enzymes. This work proposed a multiple-biomarker strategy for quality assessment of botanical drugs, while demonstrating for the first time the feasibility of microfluidics in this field.

## Introduction

Effectiveness of herbal medicines in many ailments, especially in chronic and multifactorial diseases, has been evidently proven by medical practice for thousands of years in China and many other countries. The development of botanical drugs has also attracted tremendous amount of attention from academic, pharmaceutical industry and regulatory agency worldwide^1,2^. However, this development confronts with challenges of the heterogeneous nature of herbal preparations that contain a myriad of components with diverse structures and properties. In addition, variations in geographic origin, growth condition, agricultural practice and manufacturing process contribute to the differences in chemical composition and therapeutic effect of the final product^2–4^. As a result, quality control of botanical drugs encounters many challenges to ensure their safety, efficacy and consistency.

Similar to its indispensable role in quality control of chemical drugs, chemical testing is the prevailing approach used for botanical drugs. To achieve comprehensive control of botanical drug products, state-of-art analytical techniques including high performance liquid chromatography (HPLC), gas chromatography (GC) and mass spectrometry (MS) have been increasingly employed, as indicated by quantification of marker components for authenticity, and chemical fingerprinting for batch-to-batch consistency^5–7^. Despite of their successful applications, however, several important issues remain to be addressed, including the cost and availability of standard substances, as well as their clinical relevance. Therefore, chemical testing alone may not be sufficient to ensure quality and thus therapeutic consistency of botanical drugs if bioactive constituents are not detectable/characterized or the constituents monitored cannot account for the potency and efficacy. Consequently, there is rising interest in introducing biological assays that reflect the drug’s known or intended mechanism of action as a complement to chemical analysis^8^. These approaches can be separated into two primary categories: those that evaluate the biological consistency by similarity analysis of gene/protein expression profiling^9–11^ or biofingerprinting^12,13^; and those that directly measure bioactivity of a drug on enzymes, cells, microorganisms or animals using a single biological parameter^14–17^. However, bioassays addressing a single pharmacological activity may not fully account for the efficacy of botanical drugs. In addition, results from bioassays are generally more variable than chemical tests, and the analytical procedures are usually laborious and time-consuming. Therefore, there is a clear and urgent need to develop rapid, reliable and cost-effective bioassays for quality assessment of botanical drugs.

Since its emergence, biomarker is increasingly perceived to be an essential tool in drug development, regulation and clinical research^18,19^. It is believed that judicious biomarker use can improve many of the key steps in pharmaceutical development, including target identification, lead optimization, toxicology and clinical practice. Also, featured with (clinical) relevance and validity^20^, biomarkers offer the potential to bridge the gap between quality control metrics and clinical efficacy. Therefore, we attempt herein to extend biomarkers into the realm of botanical quality control, which can be termed “quality biomarkers”—that is, measurable biological parameters that can be evaluated as indicators of drug quality. Ideally, a quality biomarker would reflect the drug’s mechanism of action, and eventually be clinically relevant. Similar to common biomarkers, quality biomarkers can be enzymes, metabolites, genes, or even gene/protein expression profiling. From a practical perspective, however, simple, feasible and easily testable biomarkers are preferred, and testing methods stratified for ease of use, sensitivity and specificity are needed for practical applications.

**Figure 1:**
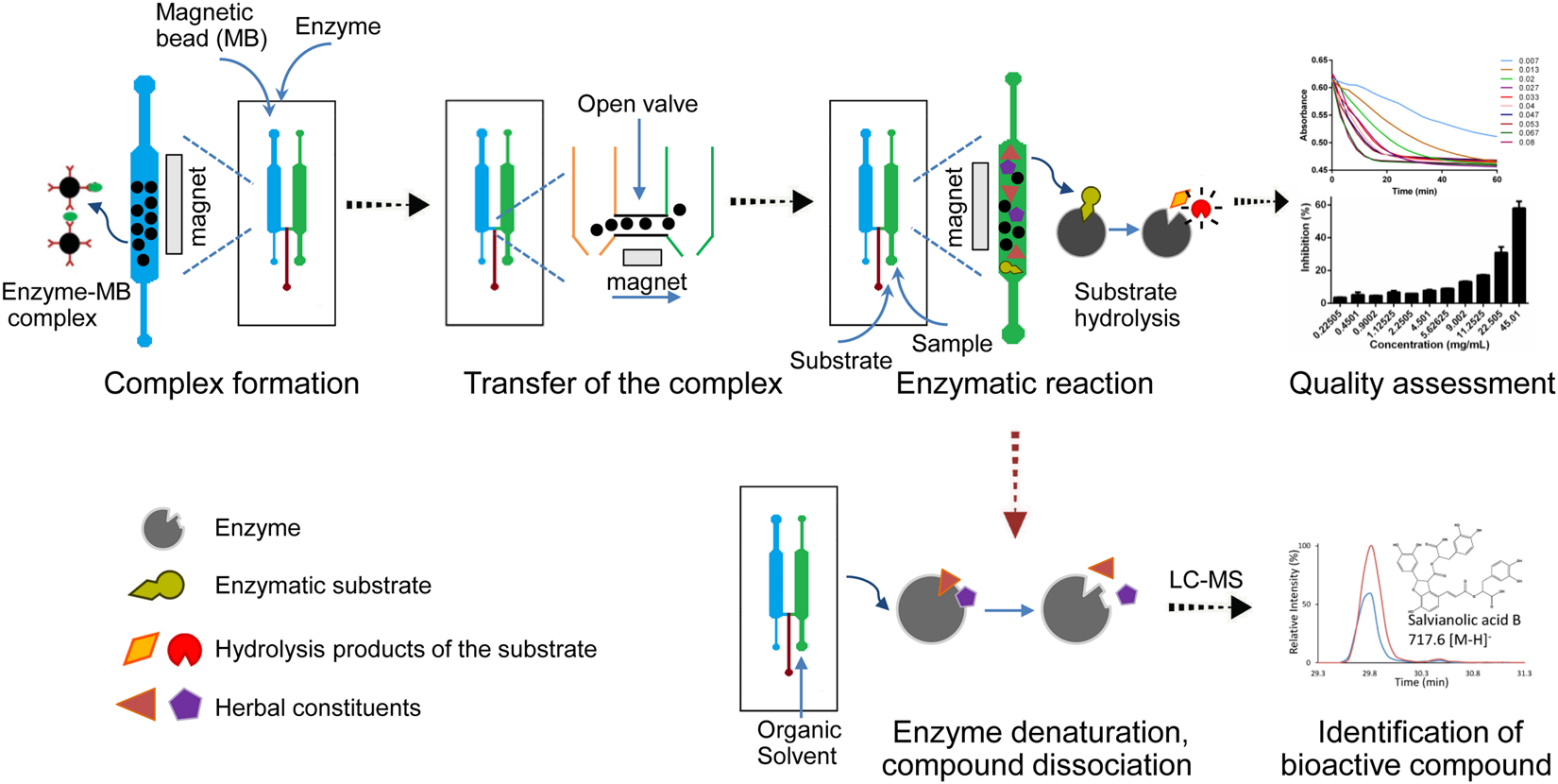
Schematic of microfluidic chip-based approach for quality assessment and screening of botanical drugs.

In this study, we proposed a chip-based approach using target enzymes as quality biomarkers to evaluate biological consistency of botanical drugs. A dual-channel microfluidic chip was designed to perform the bioassay, where one channel allowed for the formation of the enzymatic complex, and subsequent enzymatic reaction was taken place in the other channel. Magnetic beads (MBs) were employed both as an efficient surface to immobilize the enzyme and as a controllable solid support to enhance on-chip mixing and transport. Enzymatic activities were used to indicate drug quality (or potency). Moreover, this device served as a convenient screening platform for active phytochemical identification by dissociating the constituents that bound to the enzymes. The experimental workflow is outlined schematically in Fig. 1.

The utility of this approach was exemplified on QiShenYiQi Pills (QSYQ), a Chinese medicine composed of *Astragalus membranaceus* (Huangqi, HQ), *Salvia miltiorrhiza* (Danshen, DS), *Panax notoginseng* (Sanqi, SQ), and essential oil of *Dalbergia odorifera* (Jiangxiang, JX). This polyherbal preparation is widely prescribed for cardiac dysfunction, and related mechanism has been well investigated^21–23^. However, the complexity of constituents contained in QSYQ necessitates the combination of multiple analytical methods, which renders a toilsome quality control procedure: e.g., both LC and GC fingerprinting analysis is required as volatile components in JX essential oil could hardly be detected by LC^24^. Therefore, we attempted to develop an efficient quality assessment strategy for QSYQ by using quality biomarkers and microfluidics. Two enzymes, i.e. thrombin and angiotensin converting enzyme (ACE), were chosen as quality biomarkers for QSYQ according to our previous studies^23,25^ and other works^21,26^. The results demonstrate this multiple biomarker strategy can detect potency variations with good discrimination ability, reproducibility and stability, which highlights its great application potential in quality assessment of botanical drugs.

## Results

### Optimization of operational parameters

**Figure 2:**
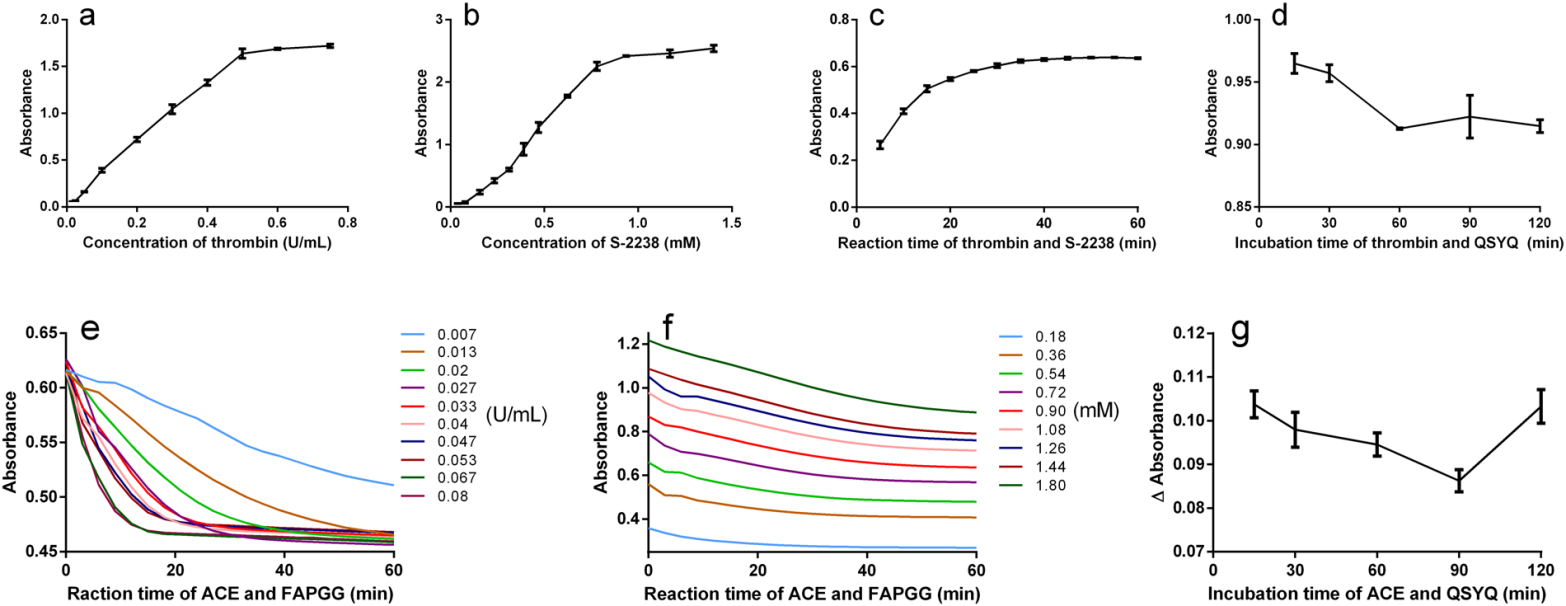
Effects of different operational parameters on the performance of the on-chip assay. (**a**) concentration of thrombin (0.29 mM S-2238), (**b**) concentration of S-2238 (0.2 U/mL thrombin), (**c**) reaction time of thrombin and S-2238 (0.2 U/mL thrombin, 0.31 mM S-2238), (**d**) incubation time of thrombin and QSYQ sample (0.2 U/mL thrombin, 0.31 mM S-2238), (**e**) concentration of ACE (0.36 mM FAPGG), (**f**) concentration of FAPGG (0.027 U/mL ACE), (**g**) incubation time of ACE and QSYQ sample (0.027 U/mL ACE, 0.72 mM FAPGG). Assays were carried out in triplicate except (**e**) and (**f**), and results were expressed as mean ± S.D. Assays for (**e**) and (**f**) were performed once, and the absorbance was measured every three minutes. For ACE assay, Δabsorbance was the absorbance decrease between 0 min and 30 min after the addition of FAPGG at 340 nm.

To enhance the performance of the on-chip assay, experimental parameters such as concentrations of enzymes and substrates, substrate reaction time, and incubation time of samples and enzymes were stepwise optimized. The activity of thrombin was determined by measuring the hydrolysis of chromogenic substrate S-2238 (HD-Phe-Pip-Arg-pNA) at 405 nm. As depicted in Fig. 2a, the absorbance increased rapidly along with the elevated concentrations of thrombin, while it reached a plateau at 0.5 U/mL. A similar trend was observed for substrate concentrations and reaction time in Fig. 2b,c, respectively. Moreover, it was clear from Fig. 2d that the maximum inhibitory effect of QSYQ sample (I1 of Table 1 in this assay) on thrombin was observed after 60 min of incubation. Therefore, to achieve favorable response and prevent signal saturation, optimal assay conditions for thrombin were determined to be 0.2 U/mL, 0.31 mM, 30 min and 60 min for the final concentrations of thrombin and S-2238, substrate reaction time and incubation time, respectively. Parameters for ACE were optimized in the same way whereas absorbance decrease (Δabsorbance) was employed because the hydrolysis product of FAPGG (N-[3-(2-Furyl)acryloyl]-Phe-Gly-Gly, substrate of ACE) had a lower absorbance than the substrate at 340 nm. Figure 2e,f display the absorbance curves obtained within 60 min for different concentrations of ACE and FAPGG, where absorbance of most samples had a steady trend after an initial descent with the reaction time. In this assay, the concentrations of ACE and FAPGG were finally set at 0.027 U/mL and 0.72 mM, where the background value was lower and the reaction time was relatively shorter (30 min). Figure 2g shows that incubation time also affects ACE inhibitory activity of the tested drug, and 90 min is most suitable for QSYQ sample.

**Table 1:** Information of QSYQ samples.

No.	Group	Batch information of raw materials				Description
		HQ	DS	SQ	JX	
I1	Intermediate	Lot 1	Lot 1	Lot 1	/^a^	Intermediates produced using different batches of HQ, DS and SQ.
I2	Intermediate	Lot 2	Lot 2	Lot 2	/	
I3	Intermediate	Lot 3	Lot 3	Lot 3	/	
I4	Intermediate	Lot 4	Lot 4	Lot 4	/	
I5	Intermediate	Lot 5	Lot 5	Lot 5	/	
I6	Intermediate	Lot 1	Lot 1^b^	Lot 1	/	I6, I7 and I8 used identical raw materials with I1 but each group had one abnormal herb which was pre-extracted.
I7	Intermediate	Lot 1^b^	Lot 1	Lot 1	/	
I8	Intermediate	Lot 1	Lot 1	Lot 1^b^	/	
I9	Intermediate	Lot 1	Lot 1	Lot 1	Lot 1	I9, I10 and I11 used identical raw materials with I1 but three batches of JX were added respectively.
I10	Intermediate	Lot 1	Lot 1	Lot 1	Lot 2	
I11	Intermediate	Lot 1	Lot 1	Lot 1	Lot 3	
P1	Preparation	/^c^				Commercially available QSYQ pills whose batch numbers were 140601, 140711, 150110, 150312 and 150803, respectively.
P2	Preparation					
P3	Preparation					
P4	Preparation					
P5	Preparation					

HQ: *Astragalus membranaceus*; DS: *Salvia miltiorrhiza*; SQ: *Panax notoginseng*; JX: essential oil of *Dalbergia odorifera*. ^a^This herb was not contained in the intermediate. ^b^The herb used in the intermediate was extracted prior to the assay. ^c^Not provided.

### Bioassays of QSYQ intermediates, preparations and extracts of single herb

**Figure 3:**
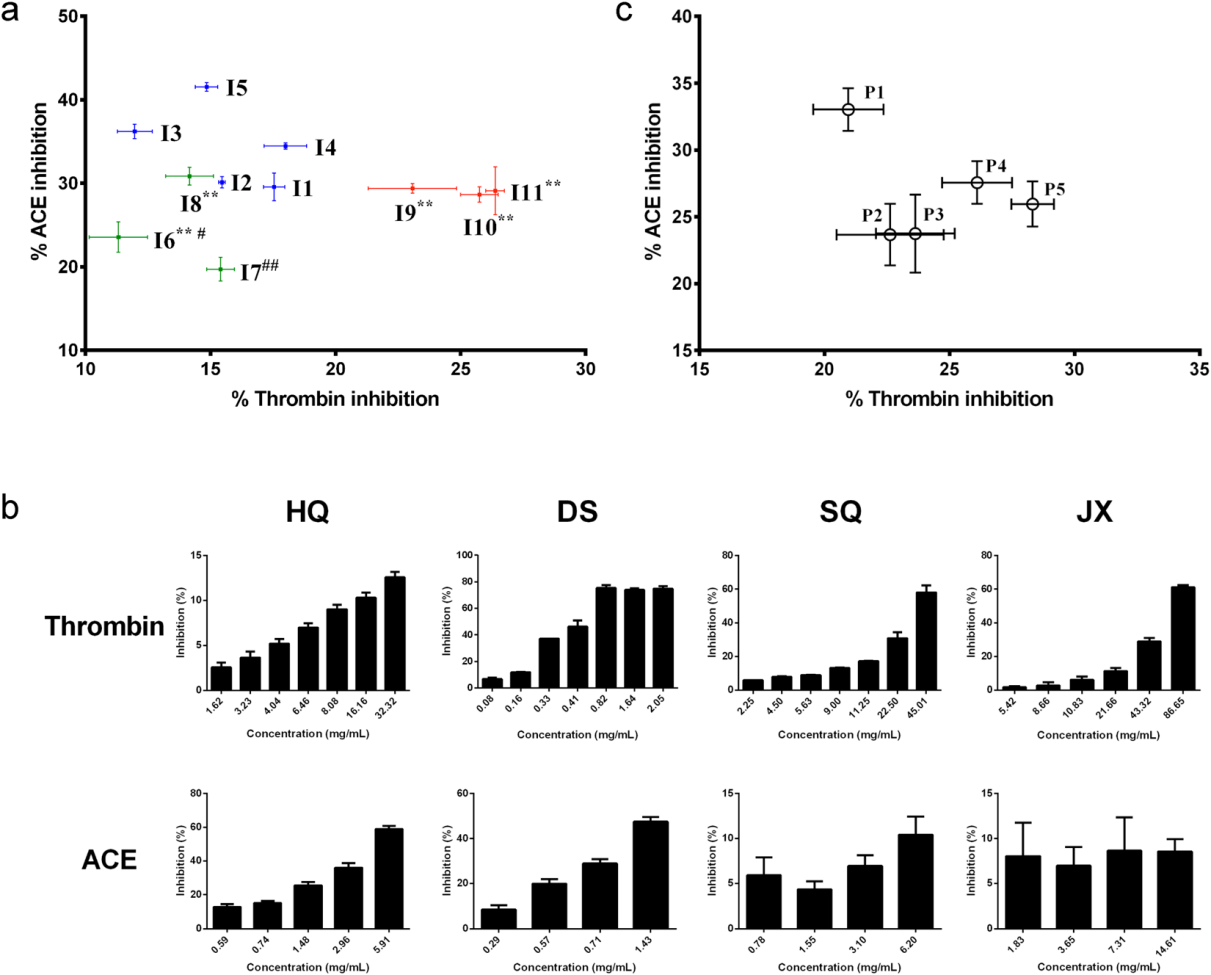
The bioassay result for QSYQ intermediates, preparations and single herbs. (**a**) thrombin and ACE inhibitions of 11 batches of QSYQ intermediates, (**b**) thrombin and ACE inhibitions of different herbs, (**c**) thrombin and ACE inhibitions of five batches of QSYQ preparations. I1 to I5 were intermediates produced using different batches of HQ, DS and SQ; I6 to I8 used identical raw materials with I1 but each group had one abnormal/pre-extracted herb; I9 to I11 used identical raw materials with I1 but three batches of JX were added respectively. HQ: *Astragalus membranaceus*; DS: *Salvia miltiorrhiza*; SQ: *Panax notoginseng*; JX: essential oil of *Dalbergia odorifera*. Assays were carried out in triplicate, and inhibition results were expressed as mean ± S.D. One-way analysis of variance (ANOVA) with Dunnett’s test was performed to compare enzyme inhibitions between I1 and I6-I11, as they were produced using the same batches of raw materials. ^**^ *P* < 0.01 compared to I1’s thrombin inhibition; ^#^ *P* < 0.05, ^##^ *P* < 0.01 compared to I1’s ACE inhibition.

The optimized protocol was first applied to assess the quality of 11 batches of QSYQ intermediates (Table 1). Enzyme inhibition (in percentages) was employed as an indicator of drug quality and calculated according to the following equations: thrombin inhibition (%) = [1 − (A_QSYQ_/A_blank_)] × 100; ACE inhibition (%) = [1 − (ΔA_QSYQ_/ΔA_blank_)] × 100; where A_QSYQ_ and A_blank_ were the absorbance of the test sample and the blank sample after the enzymatic reaction (the background value was subtracted), and ΔA_QSYQ_ and ΔA_blank_ were the absorbance decrease between 0 min and 30 min of the samples. A scatter plot was generated to display the inhibition result of thrombin and ACE simultaneously. As shown in Fig. 3a, I1 to I5, which were produced using different batches of raw herbal materials (without JX), showed similar inhibitions of both enzymes. Moreover, artificial abnormal samples I6, I7 and I8, each with one abnormal (pre-extracted) herb, presented relatively lower inhibitions compared with I1. Interestingly, influence of abnormal herbs on enzymatic activity was different. For example, I8, which contained pre-extracted SQ, showed a reduced thrombin inhibition and a similar ACE inhibition compared with I1, indicating the inhibitory effect of SQ on thrombin. Also, change of DS in I6 resulted in obvious decrease in both thrombin and ACE inhibition, whereas change of HQ in I7 imposed a greater impact on ACE than on thrombin. In addition, when three batches of JX essential oil were separately added to I1, the resulting samples, namely I9, I10 and I11, exhibited improved thrombin inhibitions and similar ACE inhibitions compared with I1, suggesting the thrombin inhibitory activity of JX.

The result indicated that inhibitions of the herbs on the enzymes were selective. Therefore, a subsequent bioassay was conducted to determine dose-response relationships between the enzymes and the herbs. As illustrated in Fig. 3b, enzyme inhibitions of different herbs varied considerably. HQ showed a weak inhibition of thrombin, while an obvious ACE inhibition of this herb was observed at 5.91 mg/mL. DS exerted potent inhibitions on both thrombin and ACE where the inhibition rate of thrombin reached approximately 80% at 0.82 mg/mL. In contrast, SQ and JX exhibited inhibitions of thrombin, whereas their inhibitions of ACE were quite weak at tested concentrations. It was clear from the result that potency of each herb could be demonstrated by at least one enzyme, so by combining the two enzymes together, the quality of QSYQ pills could be better assessed.

Five batches of QSYQ preparations (Table 1) were also analyzed by the approach. The tested samples exhibited similar inhibition potency on both thrombin and ACE except P1, for which a relatively weak thrombin inhibition and a relatively strong ACE inhibition were observed (Fig. 3c). This result suggested that there existed biological variations among different batches, and the present bioassay could evaluate such batch potency.

### Chromatographic fingerprinting analysis of QSYQ intermediates

**Figure 4:**
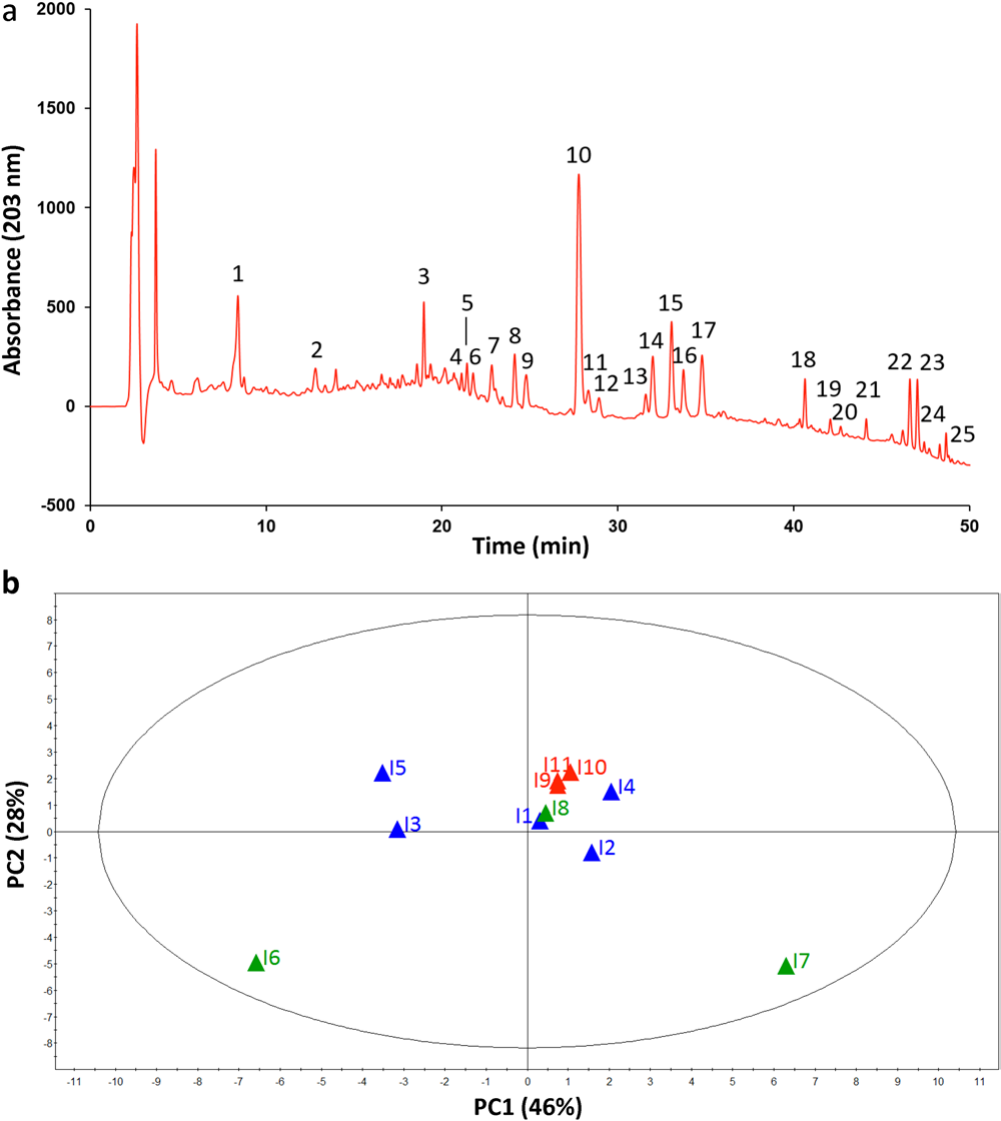
Fingerprinting analysis of QSYQ intermediates. (**a**) A representative chromatogram of QSYQ intermediate obtained by HPLC-VWD at 203 nm. (**b**) A scores plot of PCA of 11 batches of QSYQ intermediates. PCA was performed using the peak areas of the numbered peaks in the chromatographic fingerprints. Peak 3, 11, 14, 16, 17, 22 and 23, from HQ (*Astragalus membranaceus*). Peak 1, 2, 4, 5, 6, 7, 9, 10, 12, 13 and 15, from DS (*Salvia miltiorrhiza*). Peak 8, 18, 19, 20, 21, 24 and 25, from SQ (*Panax notoginseng*).

For a comparative study, chromatographic fingerprints of the 11 batches of intermediates were generated using high performance liquid chromatography-variable wavelength detector (HPLC-VWD). The VWD wavelength was set at 203 nm, and a representative chromatogram was shown in Fig. 4a. Among all the detectable peaks, those with favorable response and good resolution were chosen as common peaks to assess the similarity among samples. As a result, 25 peaks were selected, including 7 from HQ, 11 from DS and 7 from SQ, which contributed approximately 80% of the total peak area. It was noteworthy that there were no peaks from JX detected in this chromatogram. A commonly-used multivariate statistical technique—principal component analysis (PCA) was then employed to quantitatively characterize differences and similarities between the fingerprints of the QSYQ intermediates. The clustering result (Fig. 4b) resembled to some extent that of the bioassay (Fig. 3a): I1 to I5 were clustered, and I6 and I7 could be differentiated from I1. However, it was clear that the fingerprinting failed to distinguish I8 to I11 from I1, whereas these samples could be properly grouped in the biological space. A plausible reason for the proximity of I8 to I1 in PCA plot was that constituents (saponins) from SQ only presented a weak absorption at 203 nm, leading to relatively minor contribution to the separation in the scores plot. Moreover, under the current chromatographic condition, volatile compounds from JX essential oil contained in I9, I10 and I11 could hardly be detected due to their low polarity and poor ultraviolet absorption. Therefore, PCA based on chemical fingerprinting could not separate the samples into distinct clusters. The result also indicated that compared with the chemical testing, the multiple biomarker assay provided better discrimination ability for the abnormal samples.

### Screening and identification of thrombin and ACE inhibitors

**Figure 5:**
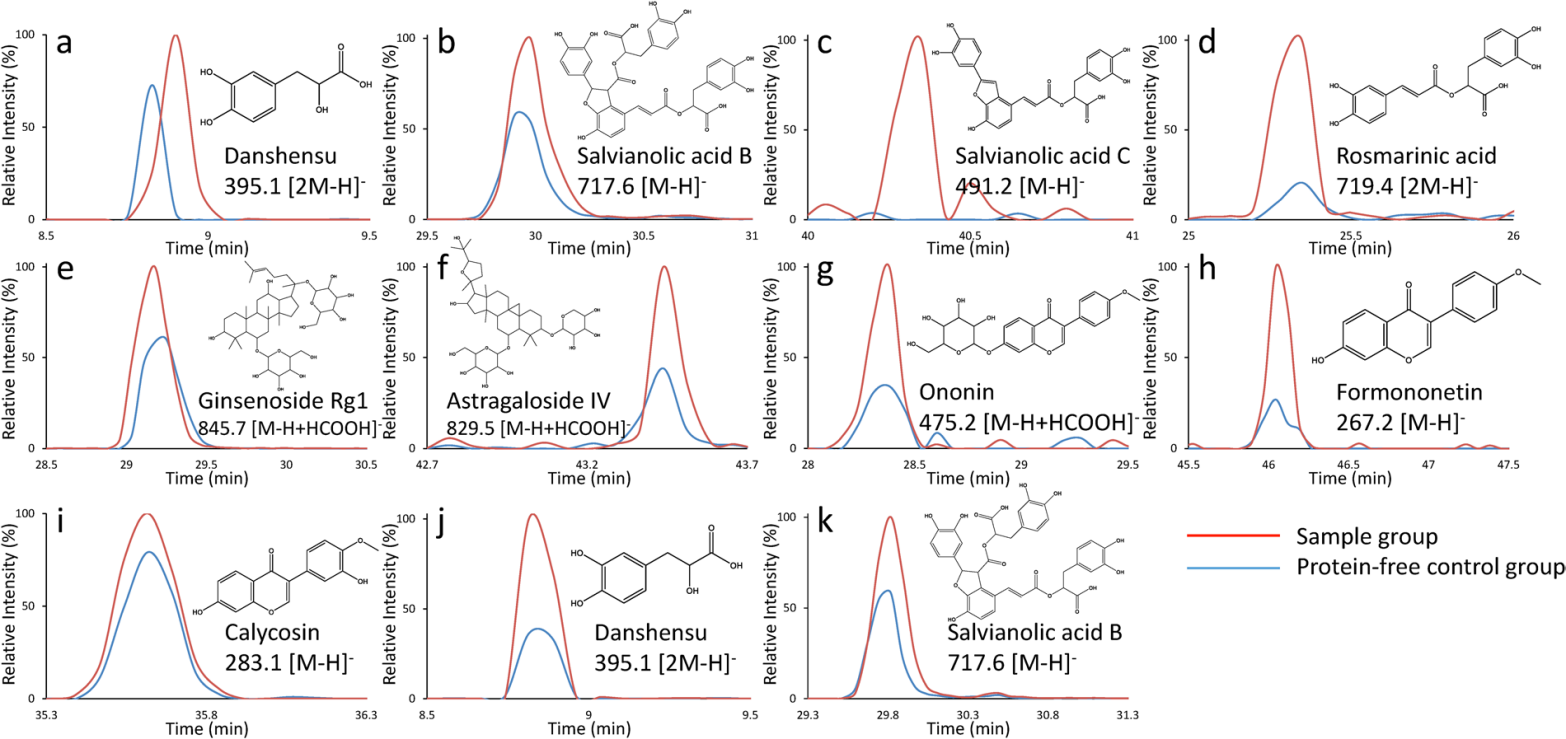
The extracted ion chromatograms (red line, sample group; blue line, protein-free control group) and the chemical structures of potential thrombin (**a**–**e**) and ACE (**f**–**k**) inhibitors identified from QSYQ. Peak area enhancements of the compounds compared with the control indicated specific binding to enzymes. The chromatograms were extracted by *m/z* shown in the figure.

In addition to quality assessment, the microfluidic chip can also function as “affinity chromatography” to identify bioactive constituents from botanical drugs in a convenient and efficient manner. After the bioassay, the bound phytochemicals were dissociated from the enzymes and accurately identified by LC-MS. The material used for the chambers in microfluidic system, polydimethylsiloxane (PDMS), is apt to adsorb small molecules nonspecifically^27^, thus a protein-free control was applied to reflect the degree of nonspecific binding of compounds to the chip. Peak areas were obtained from the extracted ion chromatography (EIC) in an ion-trap mass spectrometry (IT-MS), and binding degree (binding degree % = [(As − Ac)/As] × 100, where As and Ac were the peak area in the sample and control, respectively) was calculated to indicate binding specificity. Finally, the areas of 11 peaks were found to be significantly enhanced compared with the control (Fig. 5), suggesting constituents corresponding to these peaks were potential thrombin or ACE inhibitors with specific binding to the enzymes. All these peaks were unambiguously confirmed via comparisons with chemical standards in terms of retention time and mass spectra (Table 2), and five thrombin inhibitors (danshensu, salvianolic acid B, salvianolic acid C, rosmarinic acid and ginsenoside Rg1) and six ACE inhibitors (astragaloside IV, ononin, formononetin, calycosin, danshensu and salvianolic acid B) were preliminarily identified. The use of protein-free control was proved to be essential to eliminate false positives arising from nonspecific binding because strong background signals were observed in EICs of protein-free control. Enzyme inhibition assays of these hit compounds were then performed to verify the screening results. As presented in Table 2, among the identified ligands, four showed favorable thrombin inhibitions, while two showed ACE inhibitions. Compounds showing an inhibition rate over 70% were subjected to IC_50_ measurement. Salvianolic acid B, Salvianolic acid C and rosmarinic acid exhibited potent thrombin inhibitory effects with IC_50_ of 81.9, 180.1 and 92.0 μM, respectively. This result implied that alteration in peak area could be used for rapid identification of bioactive constituents from herbal medicines, although the binding degree was not in strict agreement with the enzyme inhibition.

**Table 2:** Characterization of bound constituents and their binding degrees and inhibition activities on thrombin and ACE.

Peak No.^a^	RT (min)^b^	Identity	Molecular formula	Major fragments in IT/MS	Bindingdegree (%)	IC_50_ (μM) or inhibition rate^e^
1	8.9	Danshensu	C_9_H_10_O_5_	197.2 [M-H]^−^179.2 [M-H-H_2_O]^−^135.3 [M-H-H_2_O-CO_2_]^−^123.2 [M-H-C_2_H_2_O_3_]^−^	50.9^c^/57.2^d^	66.9%^c^/64.2%^d^
8	24.1	Ginsenoside Rg1	C_42_H_72_O_14_	845.7 [M-H + HCOOH]^−^799.8 [M-H]^−^637.9 [M-H-(Glc-H_2_O)]^−^475.9 [M-H-2(Glc-H_2_O)]^−^	20.6^c^	/^g^
9	25.3	Rosmarinic acid	C_18_H_16_O_8_	359.1 [M-H]^−^197.1 [M-H-Caffeoyl]^−^161.2 [M-H-C_9_H_10_O_5_]^−^133.2 [M-H-C_9_H_10_O_5_-CO]^−^	67.4^c^	92.0 μM^c^
10	27.8	Salvianolic acid B	C_36_H_30_O_16_	717.2 [M-H]^−^519.3 [M-H-C_9_H_10_O_5_]^−^321.2 [M-H-2C_9_H_10_O_5_]^−^295.2 [M-H-C_9_H_10_O_5_-C_10_H_8_O_6_]^−^	34.9^c^/41.3^d^	81.9 μM^c^
11	28.4	Ononin	C_22_H_22_O_9_	429.3 [M-H]^−^297.1 [M-H-C_9_H_8_O]^−^267.2 [M-H-(Glc-H_2_O)]^−^135.8 [M-H-(Glc-H_2_O)-C_9_H_8_O]^−^	52.6^d^	30.6%^d^
17	35.6	Calycosin	C_16_H_12_O_5_	283.2 [M-H]^−^239.4 [M-H-CO_2_]^−^185.2 [M-H-C_5_H_6_O_2_]^−^135.5 [M-H-C_9_H_8_O_2_]^−^	19.1^d^	/^g^
–^f^	40.3	Salvianolic acid C	C_26_H_20_O_10_	491.2 [M-H]^−^311.2 [M-H-C_9_H_8_O_4_]^−^293.3 [M-H-C_9_H_10_O_5_]^−^267.3 [M-H-C_10_H_8_O_6_]^−^	100^c^	180.1 μM^c^
–^f^	43.4	Astragaloside IV	C_41_H_68_O_14_	829.7 [M-H + HCOOH]^−^783.7 [M-H]^−^651.6 [M-H-(Xyl-H_2_O)]^−^620.8 [M-H-(Glc-H_2_O)]^−^	28.3^d^	/^g^
22	46.1	Formononetin	C_16_H_12_O_4_	267.2 [M-H]^−^252.3 [M-H-CH3∙]^−^223.3 [M-H-CO_2_]^−^135.2 [M-H-C_9_H_8_O]^−^	53.8^d^	/^g^

^a^Compounds were numbered according to Fig. 4. ^b^Retention time of the compounds in HPLC-VWD fingerprinting. ^c^For thrombin. ^d^For ACE. ^e^Compounds showing an inhibition rate over 70% were subjected to IC_50_ measurement, otherwise only the maximum inhibition rates observed in the assay were displayed. ^f^Not selected as common peaks in HPLC-VWD fingerprinting. ^g^No obvious inhibition observed.

### Method validation

The bioassay was validated in terms of linearity, precision, repeatability and stability (samples and enzymes), while the chromatographic method was validated for precision, repeatability and stability. Supplementary Table S1 summarizes the validation result, and the measurement is expressed as relative standard deviation (RSD). For the bioassay, AEBSF-HCl and captopril were used as positive controls for thrombin and ACE, respectively. The bioassay yielded satisfactory linearity in the linear range with *r* ^2^ = 0.9988 and 0.9810 for thrombin and ACE, respectively. The precision, repeatability and stability were below 15% for both enzymes, which also indicated the enzyme solutions of thrombin and ACE kept stable within three and one days, respectively. For the fingerprinting method, the variation ranges of precision, repeatability and stability for all the 25 peaks were 0.28–3.19%, 0.16–7.68% and 0.27–8.90%, respectively. In addition, the IC_50_ determined in this assay for AEBSF-HCl and captopril were 3.91 μM and 3.31 nM, the latter of which corresponded with literature data^28–30^, also indicating the data obtained herein was reliable. These results support that the current method is of reasonable reliability and is applicable to quality assessment of botanical drugs.

## Discussion

To the best of our knowledge, this work demonstrates for the first time the feasibility of microfluidics for quality assessment of botanical drugs. Compared with conventional bioassays performed in microtiter plates, the microfluidic chip employed herein features low sample consumption of only 40 μL per determination and high automation by utilizing a syringe pump. Moreover, use of magnetic beads for enzyme immobilization not only circumvents complex surface treatments of the chips, but also enables sequential quality assessment and screening of botanical drugs in a single assay. However, rapid signal saturation is observed at high sample concentrations due to strong background noise arising from components in QSYQ, resulting in narrow tested concentration ranges and variations in readout. This may be solved in further studies by leveraging enzymatic substrates with high sensitivity and specificity, such as fluorescent probes based on aggregation-induced emission (AIE)^31^ or förster resonance energy transfer (FRET)^32^.

In general, the therapeutic effect of botanical drugs is suggested to be induced by the interference of multiple constituents on various biological targets and pathways^33^. This complexity of mechanisms represents a challenge to develop biomarker assays for quality control purposes. Bioassays specific to a single pharmacological activity may not fully address the clinical relevance, while pattern-oriented biological fingerprinting based on gene or protein expression^9,34^ is also restrained by analytical cost, reproducibility and data analysis algorithms. Ideally, a quality biomarker(s) should meet the following assay requirements which include (1) correlating with drug’s known mechanism of action and/or therapeutic effect (2) availability and testability (3) low assay cost. In this study, we propose a multiple biomarker approach employing target enzymes to reflect the drug’s known mechanism of action, based on which clinically relevant specifications are developed. Previous studies have demonstrated that QSYQ exerts protection against acute myocardial ischemia via several key pathways, including complement and coagulation cascades and renin-angiotensin system^21,23,25,26^, where thrombin and ACE are two critical enzymes mediating many important biological functions and reactions^35,36^. The present result indicates that QSYQ directly inhibits the enzymes, while each component herb also shows inhibitory activity against at least one enzyme. Thus, bioassays based on the two enzymes would be more comprehensive as each herb’s potency can be monitored. Additionally, thrombin and ACE are easily available and testable, which enables the establishment of rapid and cost-effective bioassays. Consequently, the two enzymes are chosen as quality biomarkers for QSYQ. However, it remains unclear whether the drug products with similar *in vitro* potency will deliver a consistent therapeutic effect in clinic, thus further studies are necessary to evaluate the relevance degree of quality biomarkers to clinical responses. Ideally, the establishment of such biomarker assays should be designed into the drug development process, not only facilitating evaluation of clinical relevance, but also providing more flexibility for the manufacturer to make postapproval changes^2,37^.

Different from synthetic or highly purified drugs, batch-to-batch variations are known to exist in botanical drugs. As illustrated in Fig. 3 and Fig. 4, different batches of the intermediates and preparations show variations in both biological and chemical consistency, which can be detected by the bioassay and the fingerprinting, respectively. Therefore, it is of both scientific and regulatory interest to investigate whether such variations would affect the therapeutic effect of the drug. Interestingly, there is accumulating interest in developing design space, within which changes of manufacturing parameters and material attributes are considered acceptable, to ensure the quality of chemical drugs and biologics, thereby rendering a flexible regulation of product quality. Likewise, if therapeutic consistency can be demonstrated for batches of botanical drugs with chemical or biological variations, it would lay the foundation for developing design space of botanical drugs. Actually, the design space approach based on chemical constituents (e.g. contents and yields) has been used to optimize manufacturing process of botanical drugs^4,38^. However, establishing a “potency space” may result in better botanical quality control as biological assays are more clinically relevant. This strategy may also benefit their pharmaceutical processes because manufacturing of botanical drug products involves more variable factors than that of chemical drugs.

In this work, pre-extracted raw herbal materials were prepared to mimic abnormal herbs, and such anomalies were successfully identified by the multiple biomarker assay, suggesting this approach could be used as a diagnostic tool in practical applications. Moreover, the bioassay is also capable of detecting changes of JX essential oil, volatile compounds of which could hardly be detected by LC fingerprinting. However, it is noteworthy that both chemical tests and bioassays own their respective pros and cons: botanicals with identical chemical spectrum may display different biological activities if bioactive constituents are not detectable under the analytical condition, while botanicals with different chemical profiles may have the same bioactivity when phytochemicals responsible for the difference are biologically inert. Actually, the bioassay protocol herein is considered to complement, rather than to replace, the current chemical approach. For an ideal strategy towards quality control of botanical drugs, multiple methods should be integrated together, e.g., fingerprinting to monitor batch-to-batch consistency, multi-component quantification to assure strength and authenticity, and a biological assay to measure potency and activity, which is also advocated by the U.S. Food and Drug Administration (FDA)^2^.

As mentioned above, another important aspect in botanical quality control is the characterization of active constituents that contribute significantly to a drug’s intended pharmacological activity. Unfortunately, botanical drugs contain often hundreds of chemically different constituents but only a few, if not one, have been well defined, which hampers the development of high-level quality control metrics. Microfluidic-based approaches coupled with different analytical techniques have been widely used in bioactivity screening^39–41^, and herein we utilize the flexibility of the MB-based bioassay to rapidly identify bioactive constituents in QSYQ against thrombin and ACE. Although compounds bound to be the enzymes are not necessarily the active ligands to the enzymes, the present protocol offers a cost-effective and convenient approach for bioactive identification. Among the identified constituents, danshensu, salvianolic acid B, salvianolic acid C, rosmarinic acid produced good thrombin inhibitions, while ononin and danshensu showed ACE inhibitions. Some activities to the enzymes are reported for the first time, and the present finding indicates that direct enzyme inhibition is possibly one of the mechanisms responsible for their pharmacological effects, such as platelet aggregation inhibition and thrombin time reduction^42–44^. Especially, salvianolic acid B and rosmarinic acid exhibit potent thrombin inhibitory activities with IC_50_ of 81.9 and 92.0 μM, the latter of which is also similar to the value reported by a previous study^45^. Thrombin is a key serine proteinase involved in the coagulation cascade, and is considered as a therapeutic target for thromboembolic and cardiovascular diseases^35,46^. The present finding highlights that salvianolic acid B and rosmarinic acid may be investigated as potential anti-thrombin agents. Meanwhile, taking their bioactivities into consideration, these compounds may also serve as chemical markers to promote quality standard of related botanicals.

In conclusion, we have developed a multiple biomarker assay for quality assessment of botanical drugs. A versatile microfluidic device was designed to perform the bioassay, in which enzyme-MB complex formation, enzymatic reaction and screening were together integrated. Target enzymes were used as quality biomarkers to reflect a drug’s potency and thus evaluate biological consistency. Also, phytochemicals bound to enzymes were dissociated and identified by LC-MS. The proposed protocol was successfully employed to assess the quality of QSYQ intermediates and preparations by using thrombin and ACE as quality biomarkers. Moreover, four thrombin inhibitors and two ACE inhibitors were discovered from QSYQ, providing proper bioactive markers for quality standard as well as promising drug candidates. This work not only constitutes a valuable addition to the arsenal of botanical quality control but also extends the application of microfluidics. In future studies, we will continue to work on the development of high-level quality control metrics of botanicals using emerging technologies, as well as applying them in process optimization and manufacturing control.

## Methods

### Materials and reagents

Thrombin (from human plasma), ACE (from rabbit lung), FAPGG and captopril were purchased from Sigma-Aldrich (St. Louis, USA). S-2238 was obtained from Aglyco (Beijing, China). AEBSF-HCl was purchased from Selleckchem (Houston, USA). Trizma base (Tris) was obtained from Sangon Biotech (Shanghai, China) and dimethyl sulfoxide (DMSO) from J&K Scientific (Beijing, China). All other chemicals were purchased from Sinopharm Chemical (Shanghai, China) unless otherwise indicated. 0.2 M Phosphate buffer (PB, pH 6.5), and 50 mM Tris-HCl buffer (200 mM NaCl, pH 8.3) were prepared as assay buffers for thrombin and ACE, respectively.

N-hydroxysuccinimide (NHS)-coated magnetic nanoparticles (diameter 100–150 nm, 10 mg/mL, Elut-P012) were obtained from Enriching Biotechnology (Shanghai, China). Before use, the MBs were washed twice in phosphate-buffer saline (PBS), pH 8.0 at 4 °C according to the manufacturer’s instruction.

HPLC-grade acetonitrile and formic acid were purchased from Merk (Darmstadt, Germany) and Roe (Newark, USA), respectively. Ultra-pure water was prepared by a Milli-Q Plus water purification system (Millipore, Billerica, USA). Other reagents used were all of analytical grade.

Reference compounds, including dansensu, salvianolic acid B, salvianolic acid C, rosmarinic acid, ginsenoside Rg1, astragaloside IV, ononin, formononetin, and calycosin were purchased from Shanghai Winherb Medical Technology (Shanghai, China).

Eleven batches of QSYQ intermediates (no excipients), five batches of QSYQ Pills (Table 1), extracts of HQ, DS and SQ, and JX essential oil were supplied by Tasly Holding Group (Tianjing, China).

### Sample preparation

Five grams of QSYQ Pills were ultrasonically extracted with 40 mL 70% aqueous methanol (v/v) for 40 min and then centrifuged at 10,000 rpm for 10 min to remove particles. The filtrate was evaporated to dryness in vacuum at 50 °C and the residue was stored at 4 °C before use. For bioassays, accurately weighted residues, intermediates or extracts were dissolved in assay buffer, 0.1% DMSO; for chemical tests, samples were reconstituted in 10% aqueous methanol and filtered through a 0.45 μm film before HPLC analysis.

### Design and fabrication of the microfluidic device

The microfluidic device consisted of three overlaid layers, including a top PDMS fluidic layer for fluidic control, a middle PDMS actuation layer for the physical separation of the fluidic channels, and a bottom glass slide serving as a seal. The fluidic layer was composed of two parallel channels linked by a fluidic bridge, whose opening was controlled by a deflectable membrane in the actuation layer as developed by Studer *et al*.^47^. With this design, the formation of enzymatic complexes and the enzymatic reaction could be separated thus considerably limiting cross-contamination especially that from phytochemicals. This chip was designed using computer-aided design (CAD) software and fabricated by photolithography. Briefly, SU-8 2075 photoresist (MicroChem, USA) was coated on silicon wafers, and exposed through a mask aligner (Wenhao Chip, China) with UV-light after a baking step. The patterns were then developed to create negative master molds for the fluidic and actuation layer, respectively. PDMS (Sylgard 184, Dow Corning) was prepared by mixing the elastomer and the curing solutions in a 10:1 ratio, and poured on their respective masters followed by precuring at 85 °C for 30 min to form two PDMS layers. The layers were then released from its masters, carefully aligned and bonded by oxygen plasma treatment. Finally, this bi-layer assembly was sealed on a glass slide also by oxygen plasma treatment, and the inlets and outlets were punched out. The dimensions of the fluidic channel were 10 mm long, 4 mm wide and 0.5 mm high, featuring a volume of 20 μL. The channels were primed with ethanol to prevent the formation of air bubbles before use. A photo of the microfluidic device with geometry parameters are presented in Supplementary Fig. S1.

### Protocol for on-chip botanical quality assessment and screening

MBs (10 mg/mL) were diluted 10 times in PB or Tris-HCl buffer, and 10 μL of the diluted MBs (1 mg/mL) were injected into one channel of the chip using a syringe infusion pump (Harvard Apparatus, USA) and retained by an external magnet. Then, 10 μL of thrombin or ACE solution was added and incubated with MBs for two hours at room temperature to form enzyme-MB complexes. The magnet was manually controlled to move MBs back and forth to enhance the ratio of reaction. Subsequently, MBs were washed twice in assay buffer and magnetically transferred to the other channel through the fluidic bridge, whose opening and closing was controlled by a pneumatic valve. Next, 10 μL of sample solutions of tested drug and 10 μL of enzymatic substrate were sequentially infused to the channel by the pump and incubated with the enzyme. When the enzymatic reaction was finished, the MBs were retained in the channel, and air was injected into the channel from the inlet using a syringe to push all the reacted solution to a 384-well plate. The absorbance of the solution was then monitored at 405 nm (for S-2238) or 340 nm (for FAPGG) using an Infinite F200 spectrophotometer (Tecan, Männedorf, Switzerland). Moreover, after detection, the reaction channel was washed three times by assay buffer to remove unbound constituents. 20 μL of 80% aqueous methanol was then injected and incubated with MBs for one hour to dissociate the bound constituents. The resulting solutions of replicates were combined, evaporated by a centrifugal evaporator, reconstituted in 80% aqueous methanol, centrifuged at 10,000 rpm for 10 min, and analyzed by LC-MS for chemical identification.

### Instrumentation for chemical tests

An Agilent 1100 HPLC system (Agilent Technologies, Waldbronn, Germany) coupled with an LCQ Deca XP^plus^ IT-MS (Thermo Finnigan, San Jose, CA, USA) equipped with an electrospray ionization (ESI) interface was employed for structure elucidation of the bounded constituents. The analysis was performed in negative ionization mode under following operation parameters: scan range m/z 100–1500; capillary voltage −15 V; source voltage −3 kV; capillary temperature 350 °C; sheath gas (N_2_) 60 arb; auxiliary gas (N_2_) 20 arb.

An Agilent 1100 HPLC system coupled with a variable wavelength detector (VWD) was used for fingerprinting analysis. The VWD wavelength was set at 203 nm.

The analytical condition was adapted from a previous study in this laboratory (unpublished work) with slight modifications: chromatographic separation was achieved on a Zorbax SB-C18 column (1.8 μm, 4.6 mm × 100 mm, Agilent) at 30 °C with mobile phase A (0.1% formic acid–water) and mobile phase B (acetonitrile). The flow rate was 0.4 mL/min and a linear gradient elution was programmed: 0–15 min, 5–23% B; 15–25 min, 23–24% B; 25–40 min, 24–40% B; 40–44 min, 40–58% B; 44–55 min, 58–58% B; 55–60 min, 58–95% B; 60–66 min, 95% B. The injection volume was 15 μL for both HPLC-IT/MS and HPLC-VWD.

### Method validation

To ensure the proposed test could perform in a reproducible and predictable manner, both the biological assay and the chemical test were appropriately validated in accordance with guidelines of the U.S. FDA^48–50^. The bioassay was validated in terms of linearity, precision, repeatability and stability (samples and enzymes), while the chromatographic method was validated for precision, repeatability and stability. P1 and I1 were chosen as the quality control (QC) sample for the biological and fingerprinting assay, respectively. Precision was estimated by analyzing six replications of the QC sample. Six replicates of the QC sample were prepared and analyzed to measure repeatability. For stability evaluation, the QC sample was stored at ambient temperature and then analyzed at 0, 2, 4, 8, 12 and 24 h, respectively. Enzyme solutions were kept at 4 °C and analyzed within 24 h and over three consecutive days to evaluate the stability of ACE and thrombin, respectively.

### Data processing and statistical analysis

Raw data of HPLC-IT/MS and HPLC-VWD were processed by Xcalibur 2.1 (Thermo Fisher, USA) and ChemStation B.04.02 (Agilent Technologies, USA), respectively. IC_50_ values were calculated with Prism 6.0 (Graphpad, San Diego, CA). Principal component analysis (PCA) was performed with SIMCA-P 12.0.0.0 (Umetrics, Umeå, Sweden). One-way analysis of variance (ANOVA) with Dunnett’s test was performed in PASW Statistics 18.0 (SPSS Inc. Chicago, USA).

## Electronic supplementary material

Supplemental information
