# Supplementary material for: A multiple biomarker assay for quality assessment of botanical drugs using a versatile microfluidic chip
Source: Sci Rep. 2017 Sep 25;7:12243. doi: 10.1038/s41598-017-12453-w (PMC5612938; doi:10.1038/s41598-017-12453-w)
Supplement: Supplementary file 1 — Supplemental information [file 41598_2017_12453_MOESM1_ESM.pdf]

Supplemental information for

A multiple biomarker assay for quality assessment of botanical drugs using a versatile microfluidic chip

Zhen-Hao Li[1,*], Ni Ai[1], Lawrence X. Yu[2], Zhong-Zhi Qian[3] & Yi-Yu Cheng[1,*]

[1]Pharmaceutical Informatics Institute, College of Pharmaceutical Sciences, Zhejiang University, Hangzhou, China.

[2]Center for Drug Evaluation and Research, Food and Drug Administration, Silver Spring, USA.

[3]Tianjin State Key Laboratory of Modern Chinese Medicine, Tianjin University of Traditional Chinese Medicine, Tianjin, China.

[*]Correspondence and requests for materials should be addressed to Y.-Y.C. (email: chengyy@zju.edu.cn) or Z.-H.L. (email: zhenhao@zju.edu.cn).

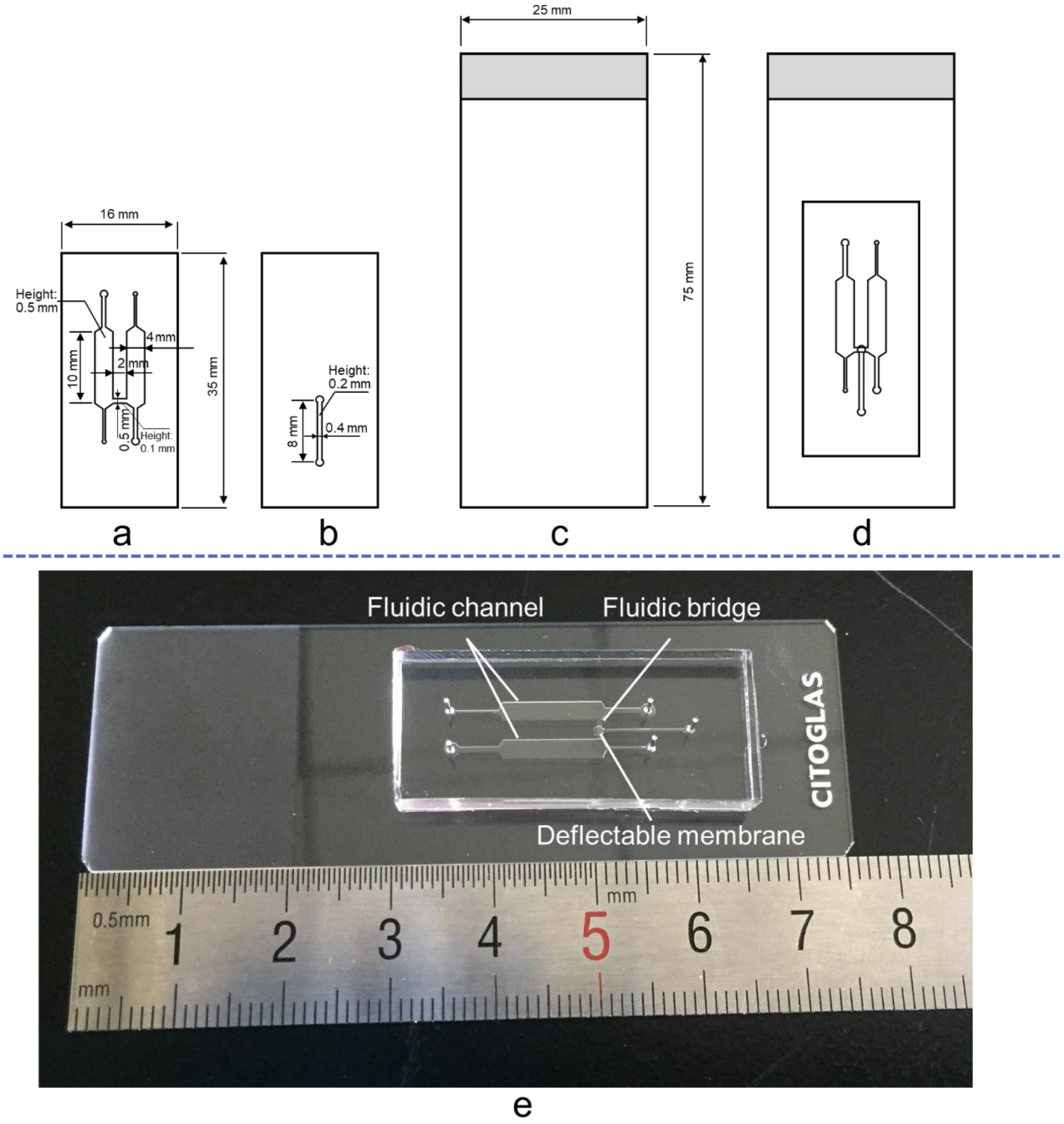

Figure S1. Structure and geometry parameters of the microfluidic chip. (a) the top PDMS fluidic layer, (b) the middle PDMS actuation layer, (c) the bottom glass slide, (d) the PDMS layers bonded on the glass slide, (e) photograph of the microfluidic chip.

No.	Precision	Repeatability	Stability (sample)	Stability (enzyme)	Linear range (mg/mL)	r^2
Bioassay						
Thrombin	10.76	13.45	13.09	8.72[a]	6.00-60.00	0.9988
ACE	6.52	5.63	8.14	7.19[b]	1.27-12.73	0.9810
Chromatographic method						
1	0.65	3.09	0.49			
2	1.11	1.18	0.73			
3	0.64	1.06	0.51			
4	0.73	4.97	0.44			
5	0.49	2.43	0.69			
6	0.71	0.56	0.57			
7	0.76	1.35	0.43			
8	1.36	2.09	1.83			
9	0.64	7.68	0.49			
10	0.28	0.85	0.27			
11	3.19	1.52	8.90			
12	1.04	1.06	5.58			
13	3.12	1.20	7.39			
14	1.27	0.59	1.19			
15	0.69	0.88	0.39			
16	0.58	2.38	0.41			
17	0.96	0.56	0.82			
18	0.52	0.45	0.33			
19	0.69	0.59	0.29			
20	0.39	1.16	0.44			
21	0.77	1.20	0.50			
22	0.61	0.16	0.41			
23	0.57	0.52	0.35			
24	0.55	0.54	0.52			
25	0.51	0.89	0.37			

Table S1. Precision, repeatability, stability (RSD%, *n*=6) and linearity of the bioassay and the chromatographic method. [a]Data acquired within three consecutive days. [b]Data acquired within one day. RSD (relative standard deviation, c_v) is defined as the ratio of the standard deviation σ to the mean μ, which are calculated by the following equations: $\mu=\frac{\sum_{i=1}^{n} x_i}{n}$; $\sigma=\sqrt{\frac{1}{n}\sum_{i=1}^{n}(x_i-\mu)^2}$;

$$c_v=\frac{\sigma}{\mu}.$$
